# Comprehensive Identification and Bread-Making Quality Evaluation of Common Wheat Somatic Variation Line AS208 on Glutenin Composition

**DOI:** 10.1371/journal.pone.0146933

**Published:** 2016-01-14

**Authors:** Huiyun Liu, Ke Wang, Lele Xiao, Shunli Wang, Lipu Du, Xinyou Cao, Xiaoxiang Zhang, Yang Zhou, Yueming Yan, Xingguo Ye

**Affiliations:** 1 Institute of Crop Science, Chinese Academy of Agricultural Sciences, Beijing, 100081, China; 2 Key Laboratory of Genetics and Biotechnology, College of Life Science, Capital Normal University, Beijing, 100048, China; 3 Institute of Vegetables and Flowers, Chinese Academy of Agricultural Sciences, Beijing, 100081, China; 4 Crop Research Institute, Shandong Academy of Agricultural Sciences, Jinan, Shandong, 250100, China; 5 Institute of Agricultural Sciences of Lixiahe Districts, Yangzhou, Jiangsu, 225007, China; Institute of Genetics and Developmental Biology, CHINA

## Abstract

High molecular weight glutenin subunits (HMW-GSs) are important seed storage proteins in wheat (*Triticum aestivum*) that determine wheat dough elasticity and processing quality. Clarification of the defined effectiveness of HMW-GSs is very important to breeding efforts aimed at improving wheat quality. To date, there have no report on the expression silencing and quality effects of *1Bx20* and *1By20* at the *Glu-B1* locus in wheat. A wheat somatic variation line, AS208, in which both *1Bx20* and *1By20* at *Glu-B1* locus were silenced, was developed recently in our laboratory. Evaluation of agronomic traits and seed storage proteins by sodium dodecyl sulfate polyacrylamide gel electrophoresis (SDS-PAGE) and reversed-phase high performance liquid chromatography (RP-HPLC) indicated that AS208 was highly similar to its parental cultivar Lunxuan987 (LX987), with the exception that the composition and expression of HMW-GSs was altered. The *1Bx20* and *1By20* in AS208 were further identified to be missing by polymerase chain reaction (PCR) and quantitative real-time RT-PCR (qRT-PCR) assays. Based on the PCR results for *HMW-GS* genes and their promoters in AS208 compared with LX987, *1Bx20* and *1By20* were speculated to be deleted in AS208 during *in vitro* culture. Quality analysis of this line with Mixograph, Farinograph, and Extensograph instruments, as well as analysis of bread-making quality traits, demonstrated that the lack of the genes encoding 1Bx20 and 1By20 caused various negative effects on dough processing and bread-making quality traits, including falling number, dough stability time, mixing tolerance index, crude protein values, wet gluten content, bread size, and internal cell structure. AS208 can potentially be used in the functional dissection of other HMW-GSs as a plant material with desirable genetic background, and in biscuit making industry as a high-quality weak gluten wheat source.

## Introduction

As one of the most important grain crops, most common wheat (*Triticum aestivum*) produced is consumed as food. The varieties and characteristics of proteins in the grain kernel are known to be critical for the food processing character of wheat flour [1]. According to their function, proteins in wheat grain can be divided into two main types: lipid metabolism proteins (LMP) and storage proteins (SP). Lipid metabolism proteins include enzymes, albumin, and globulin, and constitute around 15% of the total protein content of wheat grains. Storage proteins include gliadins and glutenins, which constitute the remaining 85% [2]. Gliadins are classified into α, β, γ, and ω types, based on their mobility in A-PAGE, and are mainly related to dough extensibility and ductility [3–4]. Glutenins are divided into high molecular weight glutenin subunits (HMW-GS) and low molecular weight glutenin subunits (LMW-GS), accounting for 12% and 33% of the endosperm proteins, respectively; these are known to be the major determinants of gluten elasticity and strength in bread making, respectively [4–6]. Although the HMW-GS content is relatively low, it determines as much as two-thirds of the bread-making quality of a wheat flour [7–9].

Since Payne and Corfield (1979) confirmed that a HMW-GS gene family member,*1Ax1*, had a close relationship with the bread-making quality of wheat flour [10], HMW-GS has been examined extensively with functional studies and with genetics, gene cloning, and molecular marker development. It is now known that that HMW-GS is encoded by genes at *Glu-1* loci located on the long arms of chromosomes 1A, 1B, 1D, known as the *Glu-A1*, *Glu-B1* and *Glu-D1* loci, respectively [11]. Each locus carries two genes that are tightly linked together and encode a larger x-type subunit (80–88 kDa) and a smaller y-type subunit (67–73 kDa). Although theoretically every wheat variety should be able to express six HMW-GSs, most bread wheat varieties typically express three to five HMW-GSs, and these frequently differ in expression levels and composition, due to allelic variation and gene silencing [12–14]. To date, more than twenty HMW-GS-encoding genes have been cloned in wheat, and their sequences have been found to be highly conserved, with the exception that there are difference numbers of DNA repeats [6, 15–17]. Allelic genetic variations at the *Glu-1* loci are known to have significant effects on wheat quality properties. *1Dx5*, *1Dx10*, *1Ax1*, *1By8*, *1Bx13*, and *1By16* may play relatively more important roles on dough elasticity and strength than other alleles, contributing to higher quality bread; the *1Dx2*, *1Dy12* and *1Bx20* alleles are known to have weak effects on the quality of glutenins [18–21]. The wheat varieties harboring genes with these negative effects are thought to be unsuitable for bread making. Expression of additional genes encoding HMW glutenin subunits in durum wheat resulted in increased dough strength and stability [22], implying that genetic transformation technology can be used as a powerful tool for improving wheat quality. To conveniently discriminate different HMW glutenin alleles contained in different wheat cultivars, some molecular markers have been developed, including markers for *1Dx5*, *1Dy10*, *1Dx2*, *1Dy12*, 1Ax2*, *1Bx7*^*OE*^, *1Bx6*, *1By8*, *1Bx17*, *1By18*, *1Bx7*, 1By8*, *1Bx7*, *1By9*, *1Bx13*, *1By16*, *1Bx14*, *1By15*, and *1Bx20* [23–31]. By using these specific markers, Jin *et al*. (2011) evaluated 718 wheat varieties and lines from 20 countries for HMW glutenin compositions [32].

Studies have shown that the effect of wheat HMW-GS on flour processing quality is also attributable to factors such as the number and location of cysteine residues [33–34], the structure of central repeat regions, the occurrence of chain termination on the proteins, the gluten of mature kernels gluten, the distribution of multimers, and the expression levels and accumulation rates of HMW-GS during grain development [35–39]. A generally accepted view on this topic is that additional cysteines, long repeat regions, and higher expression levels of HMW-GS likely have relatively positive effects on wheat dough quality [37]. HMW-GS and LMW-GS together form an intermolecular disulfide bond, and further produce glutenin aggregates, which contribute to the market value of particular food products such as bread and noodle [18–19, 40].

Even though a few genes encoding HMW-GS, including *1Dx5*, *1Dy10*, and *1Ax1* have been identified and functionally characterized through gene over-expression in wheat by genetic transformation [41–43], the roles of most HMW-GS alleles on flour processing properties remain unclear, due to the inefficient transformation system and locating effect of the glutenin genes on the chromosomes of this crop. Therefore, the functions of some HMW-GSs on bread-making quality have been indirectly characterized by combining bacterial expression systems and small-scale dough testing methods [19, 44–46]. However, these results are often unreliable. Therefore, it is necessary to develop some wheat somatic variation silencing mutants of genes encoding HMW-GSs to test their contributions to bread-making quality more precisely.

Recently, the wheat somatic variation line AS208, in which both the *1Bx20* and *1By20* genes (encoding HMW-GS protein) were silenced, was developed from the commercial wheat cultivar Lunxuan987 (LX987) by tissue culture in our research group (unpublished). In the present study, the wheat somatic variation line AS208 was examined for its HMW-GS proportions and its soluble protein content. We also performed gene cloning and gene expression analysis targeting the possible reason of the silenced *Glu-B1* in AS208. Agronomic traits, yield, and dough quality traits were also evaluated for AS208 and control wheat grown in three locations in China that each differed in terms of climate/growing conditions. Our study indicated that the proteins encoded by *1Bx20* and *1By20* play essential roles on the bread-making quality of wheat flour and suggest that the AS208 line may be potentially useful in the biscuits industry.

## Materials and Methods

### Plant materials

The common wheat (*Triticum aestivum*) cultivar, Lunxuan987 (LX987), used for tissue culture and used as the control in this study was kindly provided by Prof. Binghua Liu of the Institute of Crop Science (ICS) of the Chinese Academy of Agricultural Sciences (CAAS). The HMW-GS compositions of LX987 are 1Dx2, 1Dy12, 1Bx20, and 1By20. Wheat somatic variation line AS208 was developed by our research group from LX987 by immature embryo culture. In AS208, the HMW-GS encoding genes *1Bx20* and *1By20* at the *Glu-B1* locus were silenced. Another common wheat line, Chinese Spring (CS), which was obtained from the National Crop Germplasm Bank at CAAS, was used as the control in the reversed-phase high performance liquid chromatography (RP-HPLC) experiments in this study.

AS208 and LX987 were planted in Beijing (BJ) in the autumn of 2012 as 20 rows with a length of 1.5 m and a width of 20.0 cm. Six immature kernels from AS208 and LX987, after flowering for 5, 8, 11, 13, 15, 17, 19, 21, 23, 26, and 29 d, were collected from the middle part of the ears and immediately frozen in liquid nitrogen. Three sampled immature kernels for each sampled time point were used to extract RNA for gene expression analysis; the remaining three immature kernels from each time point were used for the extraction of glutenin for the HMW-GS composition investigation at different developmental stages.

AS208 and LX987 were planted in BJ, Shandong (SD), and Jiangsu (JS) in 10 m^2^ plots in the autumn of 2013. At maturity, ten plants were randomly picked from the two materials for the examination of agronomic traits including plant height, spike length, spikelets per ear, grains per spike, and 1000-kernel weight (TKW). Grain yield was measured after harvest.

### Extraction of seed storage proteins

Glutenin was extracted following a previously described method, with some modifications [47–48]. Mature seed samples harvested at maturity and immature seed samples collected post anthesis for different time from AS208 and LX987 were crushed and ground in a mortar and then transferred into a 1.5-ml Eppendorf tube with 800 μl of ethanol. The samples were vortexed for 20 min and then centrifuged at 13,200 rpm for 10 min. The pellet was washed 3 times with 800 μl of 55% isopropanol, and then the sample tubes were maintained at 65°C in a water bath for 40 min and subsequently centrifuged at 13,200 rpm for 10 min. Glutenin was purified from the clean pellet by adding extraction buffer consisting of 50% isopropanol, 200 mM Tris-HCl (pH 8.0), and 1% DTT or 1.4% 4-vinylpyridine (v/v). The supernatant from the immature seed samples was used for sodium dodecyl sulfate polyacrylamide gel electrophoresis (SDS-PAGE). The supernatant from the mature seed samples were divided into two portions, one of which was subjected to SDS-PAGE analysis, the other of which was subjected to reversed-phase high performance liquid chromatography (RP-HPLC) analysis, respectively.

Three additional seed proteins, including gliadin, globulin, and albumin, were extracted from mature wheat seeds according to their solubility by previously described method [49]. After being ground into a powder in a mortar, each sample was treated with 200 μl of distilled water (for albumin extraction), 1M NaCl (for globulin extraction), or 70% ethanol (for gliadin extraction). All of the samples were centrifuged for 10 min at 13,200 rpm.

### SDS-PAGE and RP-HPLC analysis

For SDS-PAGE analysis, each 60 μl sample was mixed in a Eppendorf tube with 60μl of loading buffer (2% (w/v) SDS, 80 mM Tris-HCl (pH 8.0), 40% (v/v) glycerol, and 0.02% (w/v) bromophenol blue) following an incubation in a water bath at 65°C for 20 min centrifugation at 13,200 rpm for 5 min. Electrophoresis was performed with 12% gels on a Bio-Rad PROTEAN II XL apparatus at 15 mA for 2 h, based on previously described methods [47–50]. For RP-HPLC analysis, each 60 μl sample was combined with 40 μl of cold acetone and incubated overnight at -20°C, followed by a centrifugation at 13,200 rpm for 12 min. The pellet was air dried for 30 min, then dissolved in 20 μl of a solution containing 0.05% trifluoroacetic acid and 0.5% acetonitrile and centrifuged at 13,200 rpm for 5 min. An Agilent 1100 instrument with a Zorbax 300SB-C18 column was used [50–51]. A Tunable ultraviolet (TUV) detector was used and 10 μl sample volume was injected for analyses.

### RNA isolation and quantitative real-time polymerase chain reaction (qRT-PCR) analysis

Sixty micrograms of wheat immature seeds collected at different time points of 5, 8, 11, 13, 15, 17, 19, 21, 23, 26, and 29 d post anthesis were used as samples for total RNA extraction with TRIzol reagent (Invitrogen, USA) according to the manufacturer’s instructions. Complementary DNA (cDNA) was synthesized using a reverse transcription kit (Takara, Japan).

Traditional reverse transcription PCR (RT-PCR) and qRT-PCR were both used to evaluate the expression of *1Bx20* in the somatic variation line AS208 and the parental variety LX987 at the different time points during grain development. For traditional RT-PCR, the primer pair of M1/M2 (S1 Table), specific for *1Bx20*, was used in a 20 μl reaction mixture composed of 2 μl PCR buffer (10×), 1 μl cDNA (100 ngμl^-1^), 2 μl dNTP (2 mM), 0.8 μl primer mix (10μM), 0.6 μl MgSO4 (25mM), 0.2 μl KOD-Plus-Neo (1.0 U/μl), and 13.4 μl ddH_2_O. PCR was performed with a Bio-Rad C1000 thermal cycler (Bio-Rad, USA) at 94°C for 5 min followed by 34 cycles of 94°C for 30 sec, 60°C for 30 sec, 72°C for 30 sec, and 10 min for final extension at 72°C. For qRT-PCR, the *1Bx20* specific primers of M1/M2 were applied in a 20 μl reaction volume (SYBR PrimeScript RT-PCR Kit, Takara, Japan) containing 10 μl 2× SYBR Premix Ex Taq, 2 μl first-stand cDNA, 0.3 μl primer mix (10 μM), 0.4 μl ROX Reference DyeII and 7.3 μl ddH_2_O. The amplification was performed in a ABI PRISM 7500 Real-Time PCR System (ABI, USA) with a thermal cycling program of 95°C for 5 min, followed by 40 cycles of amplification (95°C for 5 sec, 60°C for 20 sec, 72°C for 20 sec). qRT-PCR results were analyzed by ABI 7500 software and DPS (Data Processing System; IBM, USA) for standard deviation calculations.

### Dough quality analysis

A 10-gram Mixograph (National Manufacturing, USA) was used to evaluate the functional properties of AS208 dough, based on a previously described procedure [17]. Mixograph assays and SDS-sedimentation values were conducted with three repeats, following the 54–40 AAACC method [52]. The extensogram method used was based on the ICC standard [53]. Mixograph and Farinograph analyses were performed at the ICS of CAAS according to previously-described protocols [54–55]. Bread baking experiments were carried out at the Academy of State Administration of Grain, Beijing, China, to evaluate the differences between the somatic variation line and the control line in bread making qualities. The baking procedure used the standard rapid-mix-test with 1 kg flour at 14% moisture content. Each sample was mixed and baked in three repeats.

### Statistical analysis

Data analysis was performed using SPSS for Windows, version 17 (SPSS, USA). Continuous variables were compared using Student’s t-tests. The criterion for statistical significance used was *P* < 0.05.

## Results

### Comparison of several agronomic and bread-making quality traits between AS208 and LX987

**Fig 1:**
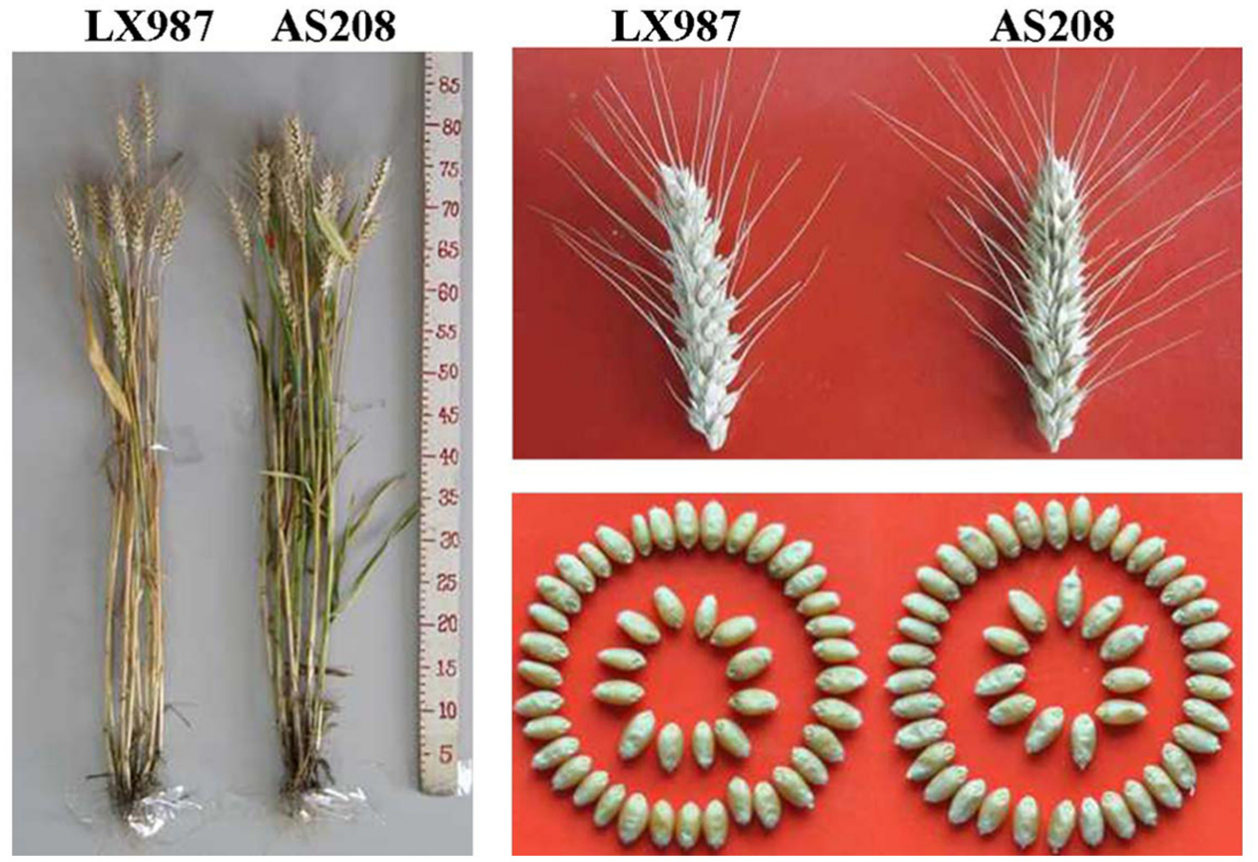
Comparison of main agronomic and seed traits between AS208 and LX987. AS208 was identical to its parental cultivar of LX987 in plant height, spike length, and grain shape.

In the second generation derived from the immature culture of LX987, a plant was found to be missing genes encoding two HMW-GSs, 1Bx20 and 1By20, at the *Glu-B1* locus. The offspring derived from this plant was sown in the autumn in a field with the ID number ‘AS208’. The seeds harvested from this line were still segregated at the *Glu-B1* locus according to SDS-PAGE (S1 Fig). The plants lacking 1Bx20 and 1By20 were selected for the next generation. Stable line AS208 was obtained from the selected plants in the fourth generation, in which the genes encoding 1Bx20 and 1By20 were completely lost (S1 Fig). To examine whether the loss of these two subunits affected the morphology of this line, AS208 was compared with LX987 in terms of several agronomic and grain quality traits with plants grown side by side in a field. AS208 and LX987 were highly similar in growth period, plant height, panicle length, spikelet number, kernels per spike, TKW, grain diameter, and water content (Fig 1, Table 1). However, a significant difference was found in grain hardness between the two lines, with the value for AS208 being much lower than that of LX987 for this trait (Table 1). These results indicated that AS208 was highly similar to LX987 in appearance and in main agronomic and grain traits, but that AS208 may have inferior traits for flour processing quality.

**Table 1:** Comparison of main agronomic and grain traits between AS208 and LX987.

Material	Plant height (cm)	Spike length (cm)	Spikelet number	TKW (g)	Grain diameter (mm)	Hardness grade	Grain water (%)
LX987	80.00±0.8	8.40±0.05	17.80±0.60	49.79±0.31	2.53±0.05	74.27(H)±0.43	11.40±0.09
AS208	80.00±1.0	8.60±0.05	20.00*±0.80	51.94*±0.25	2.62±0.04	63.90(H)*±0.38	11.20±0.11

Note: Comparison between LX987 and AS208, significance at *P* < 0.05 represented by *. The grains were harvested in Beijing (BJ) in 2013.

### Characterization of seed proteins in AS208

**Fig 2:**
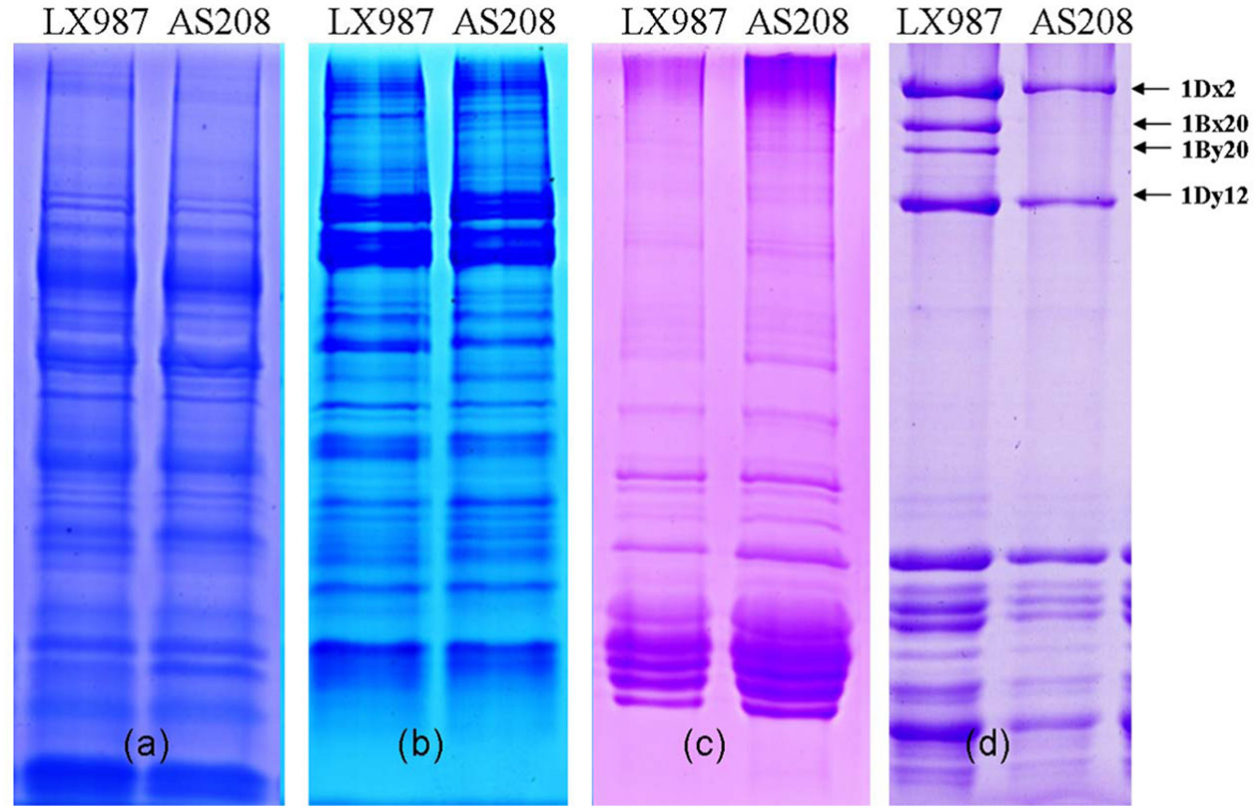
Identification of seed storage proteins in AS208 and LX987 by SDS-PAGE. The **c**ompositions for albumin (a), globulin (b), and gliadin (c) in AS208 were the same as those in its parental cultivar LX987, while the band patterns for glutenin (d) differed. The middle two HMW-GSs (1Bx20 and 1By20) were missing in the AS208 samples (d). The grains were harvested in Beijing in 2013.

**Fig 3:**
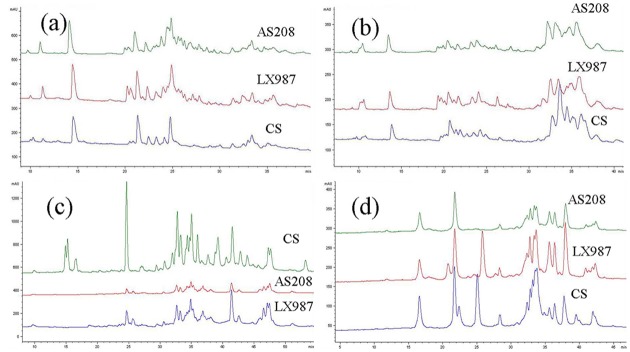
Identification of seed storage proteins in AS208, LX987, and CS by RP-HPLC. The peaks for albumin (a), globulin (b), and gliadin (c) in AS208 and LX987 were very similar, but were different from those of CS. However, the peak patterns for glutenin (d) were different among AS208, LX987, and CS, in which the three wheat accessions all had peaks for 1Dx2 and 1Dy12, while AS208 was missing peaks for 1Bx20 and 1By20 compared to its progenitor genotype LX987. The grains were harvested in Beijing in 2013.

To investigate the biochemical changes of the somatic variation line AS208 in seeds, the storage protein glutenin, gliadin, globulin, and albumin, were extracted and separated by SDS-PAGE and RP-HPLC. AS208 extracts were compared with LX987 and CS extracts. The band patterns of albumin, globulin, and gliadin in AS208 were the same as those of LX987 (Fig 2A, 2B, and 2C). The band patterns of glutenin were different in the HMW-GSs region, where the 1Bx20 and 1By20 bands were present in LX987, but missing in AS208 (Fig 2D). No difference was observed in the LMW-GSs region between the two lines. Further, the chromatography peaks for albumin, globulin, and gliadin in AS208 were identical to those in LX987, but different from those in model wheat cultivar CS (Fig 3A, 3B and 3C). In contrast, the peaks for glutenin were different among AS208, LX987, and CS. The three cultivars showed common peaks for 1Dx2 and 1Dy12 (Fig 3D). AS208 was missing the peaks of 1Bx20 and 1By20 in comparison with LX987, while CS displayed other two peaks of 1Bx7 and 1By8 (Fig 3D). We concluded that AS208 was highly similar to LX987 in seed proteins with the noted exception in the HMW-GS composition.

### Expression analysis of *1Bx20* in AS208 and LX987 during seed development

**Fig 4:**
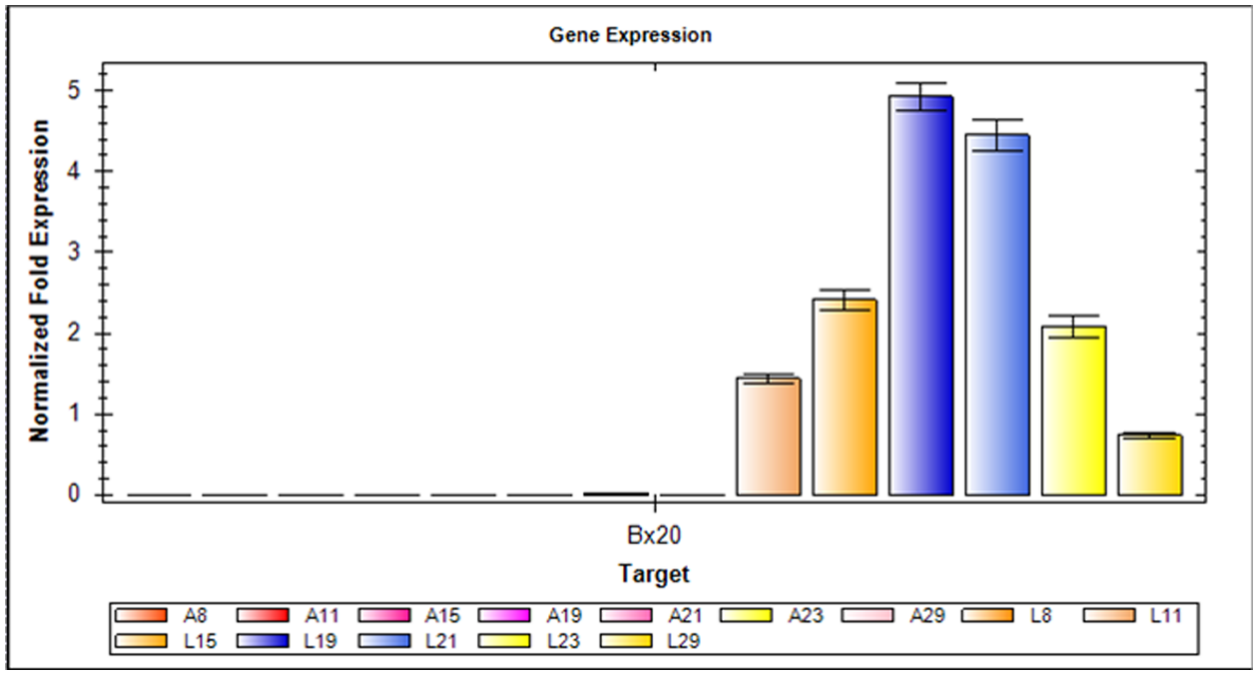
Expression profile of the *1Bx20* gene at different development stages in LX987 and AS208 assessed by qRT-PCR. The *1Bx20* gene was not expressed at any stages during grain development in AS208 (A8-A29). Its expression in LX987 started from the 11^th^ day since anthesis (L11), reached its highest level at the 19^th^ day post anthesis, and then gradually declined as filling progressed (L21-L29).

The above results confirmed that the 1Bx20 and 1By20 proteins were lost in the mature seeds of AS208. To clarify if the *1Bx20* and *1By20* genes at *Glu-B1* locus were expressed and their products could be detected in AS208 during grain development, especially at the early grain filling stage, RNA and glutenin were extracted from the immature grains of AS208 and its progenitor genotype LX987 at increasing days post anthesis. Then, the RNA and glutenin samples were analyzed by qRT-PCR and SDS-PAGE for the expression of *1Bx20* and *1By20*. We found that the expression of *1Bx20* in LX987 started from the eleventh day, reached its highest level at 19 days post anthesis, and then gradually declined as filling progressed (Fig 4). *1Bx20* was not expressed in AS208 at any point of the post-anthesis time course tested in this study (Fig 4). It should be noted that we did not attempt to measure the expression of *1By20*, because we did not develop specific primers for *1By20* due to the highly conserved sequence features of the *HMW-GS* genes. SDS-PAGE analysis showed that 1Dx2 and 1Dy12, as well as the LMW-GSs, accumulated normally in AS208 starting from the eleventh day post anthesis, while neither 1Bx20 nor 1By20 was detected in the AS208 samples during seed development (S2 Fig), which was in consistent with the qRT-PCR results (Fig 4).

### Yield and agronomic quality traits of AS208 grown in three different regions

AS208 and LX987 were planted in the autumn in Beijing, Shandong, and Jiangsu in 2013 in order to compare yield and agronomic traits. The yields of AS208 grown in the three regions were 5844.0 kg/hectare, 6733.5 kg/hectare, and 6369.0 kg/hectare, respectively. These were almost the same as the LX987 yields in the corresponding regions (Table 2). Compared with its parental variety, AS208 was equivalent in most of the external agronomic traits in the three experimental locations, including plant height, spike length, grain number per spike, and TGW (Table 2). This analysis reveals that there were no significant differences in grain yield or agronomic traits between AS208 and LX987 in differing experimental locations.

**Table 2:** Yields and agronomical traits of AS208 and LX987 grown in different experimental locations.

Material	Yield (kg/hectare)	Plant height (cm)	Spike length (cm)	Grains per spike	TGW (g)
LX987-BJ	6130.5±11.8	78.8±0.4	7.9±0.2	34.7±0.6	31.8±0.4
AS208-BJ	5844.0*±12.0	79.0±0.6	8.2±0.2	36.6±0.4	34.7*±0.5
LX987-SD	6688.5±13.2	95.0±0.8	NA	41.6±0.7	40.0±0.3
AS208-SD	6733.5±11.4	95.5±0.7	NA	37.2*±0.5	39.2±0.4
LX987-JS	6358.5±9.3	76.3±0.5	NA	40.8±0.8	36.1±0.4
AS208-JS	6369.0±10.8	77.0±0.6	NA	42.0±0.4	37.6±0.2

Note: Comparison between LX987 and AS208, significance at P < 0.05 represented by *. BJ, SD, and JS stand for the plants grown in Beijing, Shandong, and Jiangsu, respectively, which is the same in the following tables.

The grains of AS208 and LX987 harvested in the three regions in 2014 were tested for several main parameters of dough processing quality. The flour yield of AS208 in the three sites was lower than that of LX987, especially in Jiangsu, but there were no significant differences in flour moisture, ash, or crude protein content between the two lines grown in different places (Table 3). Falling number is a reflector of amylase activity in flour. The falling number of AS208 was significantly lower than that of LX987 grown in the three experimental locations (Table 3), indicating that the amylase activity was slightly higher in AS208 than in LX987. Gluten is a very important component for dough and bread quality. Gluten index is known to be closely positively associated with the strength of gluten. Both wet gluten content and dry gluten content were lower in AS208 than in LX987 in different experimental locations (Table 3). Together, these results indicate that the lack of the two HMW-GS subunits, 1Bx20 and 1By20, led to dramatic declines in the main processing quality parameters such as falling number and the wet and dry gluten content.

**Table 3:** Comparison of several quality parameters between AS208 and LX987.

Samples	Flour yield (%)	Moisture (%)	Ash content (%)	Crude protein content (%)	Falling number	Wet gluten content (%)	Dry gluten content (%)
LX987-BJ	67.61±0.42	15.35±0.04	0.69±0.03	15.49±0.01	534±4	40.2±0.4	13.7±0.1
AS208-BJ	65.12±0.31	15.15±0.05	0.66±0.04	14.58*±0.02	492*±5	34.1*±0.1	12.4*±0.1
LX987-SD	67.38±0.12	14.80±0.03	0.59±0.03	13.99±0.01	420±4	14.4±0.1	38.8±0.4
AS208-SD	66.51±0.24	15.68±0.01	0.62±0.05	14.21*±0.04	365*±3	10.6*±0.1	19.0*±0.1
LX987-JS	68.42±0.33	15.30±0.01	0.67±0.01	13.68±0.01	439±1	31.8±0.3	11.4±0.1
AS208-JS	64.67*±0.15	15.51±0.03	0.64±0.02	13.73±0.02	405*±3	29.4*±0.2	10.5*±0.1

Note: Comparison between LX987 and AS208, significance at *P* < 0.05 represented by *.

**Fig 5:**
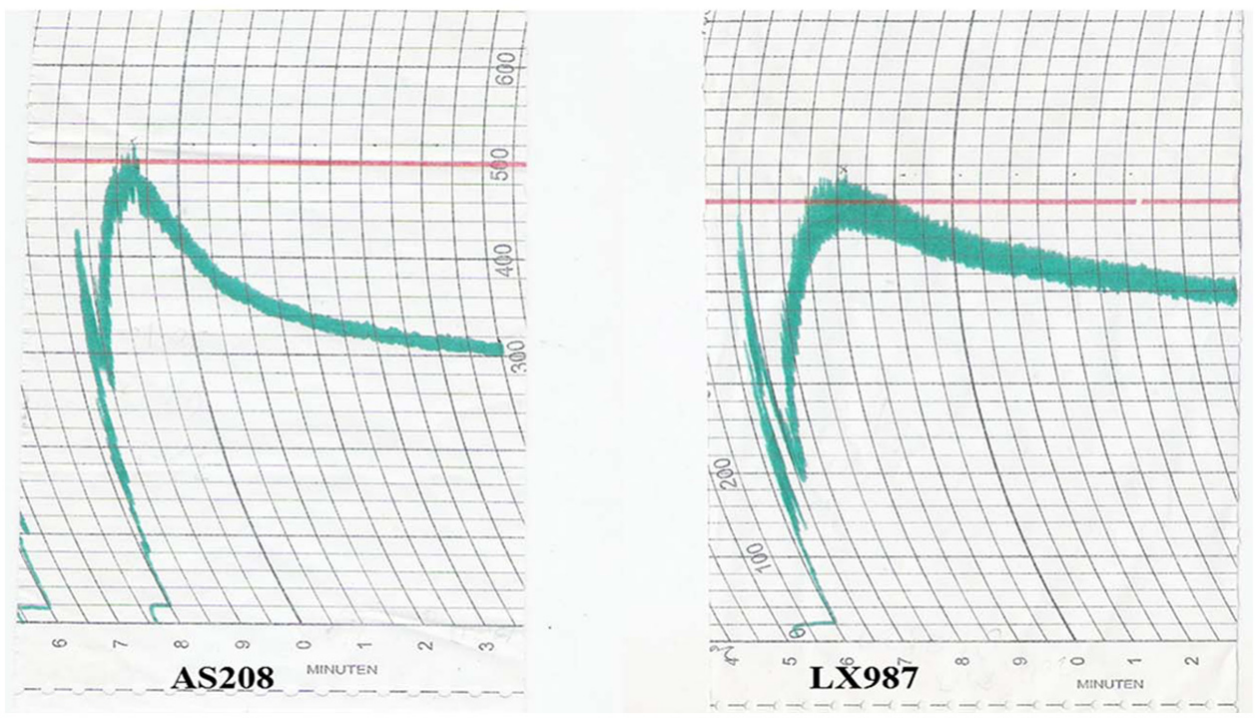
Comparison of Farinograph for AS208 and LX987. The dough formation time and the stabilization time for AS208 (left) were both shorter than the LX987 times (right) during noddle and bread processing. The grains were harvested in Beijing in 2014.

The two wheat lines produced in different locations in 2014 were analyzed with a Farinograph and a Mixograph. Even though the two accessions had similar water absorption capacity, both the dough formation time and the stabilization time of AS208 were significantly shorter than the times for LX987 (Fig 5, Table 4). In particular, the mixing tolerance index was 100 in LX987 but 200 in AS208. In addition, the flour softening degree of AS08 was increased compared with LX987 (Table 4). There was no significant difference between AS208 and LX987 in Farinograph quality number (Table 4). These results indicated that AS208, which lacks both 1Bx20 and 1By20, was classified as a weak gluten wheat, while LX987 was classified as a strong gluten wheat.

**Table 4:** Mixograph parameters of AS208 and LX987 in the three experimental locations.

Materials	Water absorption (500fu)	Formation time (min)	Stabilization time (min)	Degree of softening	Mixing tolerance index	Farinograph quality number
LX987-BJ	59.8±0.1	2.9±0.2	2.1±0.1	72.0±3.0	100±5.0	494.0±4.0
AS208-BJ	58.6*±0.1	1.6*±0.2	1.4*±0.2	163.5*±3.5	200*±8.0	502.5±2.5
LX987-SD	56.9±0.2	2.0±0.1	1.5±0.2	124.0±8	100±4.0	500.5±7.5
AS208-SD	55.8*±0.1	1.7±0.1	1.2*±0.1	166.5*±1.5	200*±4.0	500.0±2.0
LX987-JS	57.1±0.1	2.0±0.1	1.3±0.1	139.5±2.5	100±3.0	498.0±6.0
AS208-JS	55.5*±0.2	1.4*±0.1	1.3±0.3	168.5*±4	200*±7.0	502.0±8.0

Note: Comparison between LX987 and AS208, significance at P < 0.05 represented by *.

### Bread-making characteristics of AS208 in three regions

**Fig 6:**
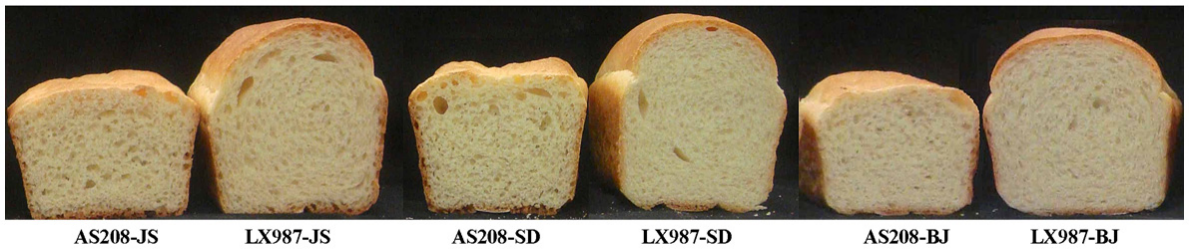
Bread shapes made from AS208 and LX987 flour from plants grown in different experimental locations. The size of bread made with flour of AS208 was much smaller than that of bread made with flour of LX987 produced in the three experimental locations. BJ, SD, and JS stand for the wheat grains harvested in Beijing, Shandong, and Jiangsu, respectively.

The grains of AS208 and LX987 harvested in Beijing, Shandong, and Jiangsu in 2014 were further tested for bread-making quality by a baking method, and several parameters of bread structure were assessed by C-Cell analysis. The results of the bread-making experiments clearly showed that the bread volume was 400–510 cm^3^ for AS208 and 600–627 cm^3^ for LX987 in the three experimental locations. AS208 was declined by 17% to 30% in bread volume compared with LX987 (Fig 6, Table 5). According to C-Cell analysis, AS208 had lower values for section area, wrapper length, slice brightness, cell contrast, stoma number, average stoma extension, and net stoma extension than did LX987 in the three experimental locations (Table 5). AS208 had higher values for cell diameter, number ratio of big and small cells, and cell wall thickness than did LX987 (Table 5). These results indicated that the processing quality and main bread-making-related parameters of AS208 became significantly worse. These results implied that 1Bx20 and 1By20 play important roles in bread-making quality in the wheat flour industry.

**Table 5:** Bread size and slices C-Cell parameters between AS208 and LX987 produced in different regions.

Materials	Bread size (cm^3^)	Slice area	Wrapper length	Slice brightness	Cell contrast	Number of cells	Cell density	Wall thickness	Cell diameter	Coarse/Fine clustering	Average cell elongation	Net cell elongation
AS208-BJ	510±5	175079±60	1580±5	132.7±0.4	0.70±0.01	1942±21	0.011096	3.50±0.01	16.26±0.21	0.205±0.022	1.46±0.01	1.05±0.02
LX987-BJ	626*±10	216639*±404	1707*±7	139.3*±0.6	0.73*±0.01	2479*±26	0.011444	3.30*±0.02	15.30±0.32	0.087±0.006	1.66*±0.01	1.22*±0.01
AS208-SD	497±12	169845±608	1573±15	129.6±0.7	0.70±0.01	1725±44	0.010158	3.60±0.03	18.47±0.01	0.236±0.018	1.46±0.02	1.09±0.01
LX987-SD	600*±5	217447*±178	1703*±12	142.0*±0.1	0.74*±0.01	2607*±29	0.011989*	3.27*±0.02	14.20*±0.31	0.105*±0.004	1.61±0.01	1.16*±0.01
AS208-JS	425±9	149046±857	1473±26	126.0±3.2	0.70±0.01	1764±3	0.011864	3.42±0.01	15.53±0.42	0.132±0.013	1.43±0.01	1.08±0.01
LX987-JS	605*±6	210162*±576	1688*±16	138.9±0.4	0.71*±0.01	2268*±12	0.010794	3.42±0.01	16.49±0.48	0.105±0.08	1.55*±0.01	1.15*±0.01

Note: Comparison between LX987 and AS208, significance at P < 0.05 represented by *.

## Discussion

### Development of wheat HMW-GS silencing mutants

Wheat seed storage proteins, especially the HMW-GSs, play very important roles in bread-making quality [6]. To date, our knowledge of the function of each particular HMW-GS is limited. It is necessary to clarify the detailed contributions of each of the HMW-GSs in bread-making quality. We are of the opinion that developing wheat near-allellic variation lines at *Glu-1* loci is an ideal strategy to achieve this purpose. Lawrence *et al*. (1988) developed seven homozygous wheat lines lacking one or two HMW-GSs at *Glu-A1*, *Glu-B1*, and *Glu-D1*, by crossing a null mutant line at the *Glu-B1* locus with an isogenic line at the *Glu-A1* and *Glu-D1* loci [56]. By using these lines, the bread-making properties of four HMW-GSs (1Dx5, 1Dy10, 1Bx17, and 1By18) were initially characterized [56]. To precisely dissect the contribution of each of the HMW-GSs to bread-making quality in the same genetic background, Yang *et al*. (2014) successfully used ion beam methods to develop three deletion lines lacking *Glu-A1*, *Glu-B1*, or *Glu-D1*, respectively. Decreased LMW-GSs content and increased accumulation of gliadins were observed in these three mutants [57]. Their research further confirmed the rank of the genetic effects of *Glu-1* loci on gluten functionality as *Glu-D1* > *Glu-B1* > *Glu-A1*, and suggested that *Glu-1* loci affected gluten functionality by promoting the formation of glutenin macropolymers (GMP) and balancing the ratios of HMW-GSs, LMW-GSs, and gliadins [57]. Most recently, Li *et al*. (2015) developed a complete set of knockout and missense mutants for 1Ax1, 1Bx14, 1By15, 1Dx2, and 1Dy12 induced by ethylmethanesulfonate (EMS) in which one or more of those above mentioned HMW-GS was knocked out, respectively, from a winter wheat variety (Xiaoyan54) [21]. By comparing bread-relevant traits of wild-type and HMW-GSs deficient single or double mutants, the functions of 1A1 and 1Bx14 on dough functionality and bread-making quality were characterized in detail [21].

Tissue culture can be used to induce variation to wheat storage protein alleles [58–59]. In regenerated plants from the immature embryo cultures of the common wheat line CS, the variation for one protein band and protein bands controlled by the locus, mostly for gliadins, were observed with frequencies of 0.11% and 0.69%, respectively [58]. In the regeneration offspring from *in vitro* culture of immature embryos of four winter varieties, one plant lacking 1Bx7 and 11By9 at the *Glu-B1* locus was found, and alteration at this locus with decreased expression occurred in only one wheat genotype [59]. In this study, we reported a somatic variation mutant AS208 lacking genes encoding 1Bx20 and 1By20 that was identified from the tissue culture offspring of a commercial winter variety, LX987. Further, we comparatively evaluated the agronomic traits, seed storage proteins, and expression pattern of *Glu-B1*, and evaluated the dough traits and bread-making quality traits of AS208 and its parental variety.

### Silencing mechanism of glutenin subunits 1Bx20 and 1By20 in AS208

Gene silencing falls into two major mechanistic classes, transcriptional gene silencing (TGS) and post-transcriptional gene silencing (PTGS) [60]. PTGS refers to the situation where the target gene is expressed normally at the mRNA level. A PTGS mechanism *1Bx20* and *1By20* in AS208 can be excluded, because our qRT-PCR result showed that *1Bx20* was not expressed in the mutant line at all (Fig 4). TGS refers to the situation where the expression of a target gene is somehow blocked because of epigenetic alternations such as chromatin modification and DNA methylation [61–62]. To investigate if the lack of 1Bx20 and 1By20 accumulation in AS208 results from TGS, two primer pairs (Px1 and Px2 for 1Bx20, Py1 and Py2 for 1By20) (S1 Table) were used to amplify the promoters of *1Bx20* and *1Bx20y* from LX987 and AS208, respectively. The promoter regions of *1Bx20* and *1Bx20y* were successfully obtained in LX987, but not in AS208. Two primers pairs (Bx1 and Bx2 for *1Bx20*, By1 and By2 for *1Bx20y*) (S1 Table) were used to amplify the open reading frame (ORF) regions of *1Bx20* and for *1Bx20y* from LX987 and AS208. Consequently, the coding regions of 2.2 kb for *1Bx20* and 2.0 kb for *1Bx20y* LX987 were obtained for LX987, but not for AS208. The amplification results for the promoters and ORFs of *1Bx20* and *1Bx20y* were consistent. Therefore, we speculate that *1Bx20* and *1Bx20y* are likely deleted in the genome of AS208. In order to further support this speculation, the specific primer pair M1 and M2 (S1 Table) for *1Bx20* were used to amplify the genomic DNA of LX987 and AS208, and the results also showed that *1Bx20* was not amplified from AS208 gDNA, but did amplify from LX987 gDNA (S3 Fig). Therefore, we suggested that the *1Bx20* and *1Bx20y* in AS208 was deleted from its genome led to their slicing in AS208. In previous reports, the silencing of genes encoding storage proteins was suggested to be caused by chromosomal deletion and promoter region alterations [58–59]. The silencing mechanism of the *1Bx20* and *1Bx20y* in AS208 needs to be investigated further at the molecular level.

### Potential Application of AS208 in the functional studies of wheat genes encoding HMW-GSs

The contributions of different individual HMW-GS proteins on gluten functionality and end-use traits have been investigated with plant materials with allelic variation for HMW-GSs, knockout mutants, bacterial expression combining small-scale dough testing system, and transgenic strategy [21, 41–45. 56–57]. Among these methods, genetic transformation is the most straightforward method for testing the function of the genes encoding HMW-GSs. To date, only a few HMW-GSs, such as 1Dx5 and 1Dy10 were functionally explored through transgenic approaches that manipulated the genes that encode these proteins. Highly efficient wheat transformation methods mediated by *Agrobacterium* have been reported by several groups in recent years [63–65]. This significant improvement provides powerful support for the reliable functional testing of genes of the *Glu-1* loci of wheat. In our previous studies, the parental wheat variety LX987 of AS208 showed available regeneration ability from mature embryos scraped into pieces and immature embryos infected by *Agrobacterium* [66–67]. AS208 can be transformed by *Agrobacterium* for obtaining transgenic wheat plants (data unpublished). Therefore, AS208 can be used as a desirable receptor material in efforts to develop transgenic lines to characterize the functions of other genes encoding HMW-GS proteins at *Glu-1* loci.

### Potential application of AS208 in flour processing industry

Wheat flour is mainly used to make bread and biscuits [1]. For the use in bread or noodle making, wheat cultivars are required to have strong gluten and high protein content. For making biscuits or cake, wheat cultivars with weak gluten and low protein level are required [6]. According to the standards for wheat quality in China (GB /T17893-1999), detailed indicators for weak gluten wheat include falling number values of more than 300 sec, crude protein content less than 11.5%, wet gluten content (at the condition 14% water level) less than 22.0%, and dough stability time less than 2.5 min. Presently in China, the number of wheat varieties that meet the standards for biscuits making is very limited (less than 20). AS208 meets the requirements for weak gluten wheat, though it does not have suitable protein or wet gluten levels (Tables 3 and 4). It can be crossed with low protein wheat varieties to develop more suitable lines for use in biscuit or cake making.

## Conclusions

In this study the genes encoding the HMW-GS proteins 1Bx20 and 1By20 at the *Glu-B1* locus in the wheat somatic variation line AS208 developed by tissue culture were found to be missing. AS208 maintained high similarity to its parental cultivar LX987 in agronomic traits and seed storage protein content, except for the HMW-GS composition. The expression of *1Bx20* and *1By20* genes in AS208 was silenced from the beginning of grain filling and remained silenced during the entire development period of grains. *Glu-B1* locus was further demonstrated to be deleted from the genome of AS208. The lack of 1Bx20 and 1By20 subunits in AS208 negatively affected dough traits and bread-making quality traits. AS208 can potentially be used to breed novel wheat varieties with weak glutenin content and to identify the functions of other genes encoding HMW-GS proteins at the *Glu-1* loci.

## Supporting Information

S1 Fig Screening of wheat somatic variation mutants at the *Glu-B1* locus in the tissue culture offspring of LX987 by SDS-PAGE. In the second generation derived from tissue culture (a), a plant was found to be missing 1Bx20 and 1By20 (sample 5). The plants from sample 5 appeared different bands for the composition of HMW-GS, some with and some without 1Bx20 and 1By20 in the third generation (b). Stable line AS208 was obtained from sample 5 in the fourth generation, in which the two bands of 1Bx20 and 1By20 were missed completely. WT stands for LX987.
(DOC)

S2 Fig Expression patterns of genes encoding glutenin subunits in AS208 at different stages during grain development assessed by SDS-PAGE-. *1Dx2* and *1Dy12*, as well as *LMW-GSs* genes expressed stably in AS208 from the 11^th^ day post anthesis (lane 3), while *1Bx20* and *1By20* were not expressed at any point during the whole period of grain development. 1–10 stand for the samples collected at 5, 8, 11, 13, 17, 19, 21, 23, 26, and 29th day post anthesis, respectively.
(DOC)

S3 Fig Detection of *1Bx20* in AS208 and LX987 with the M1 and M2 PCR markers. *1Bx20* was not detected in the genome of AS208 by the marker (nothing was amplified). However, *1Bx20* was detected from genomic DNA of LX987 by the markers (a 216-bp fragment was amplified). M represents a DL2000 DNA marker.
(DOC)

S1 Table The PCR primers used in this study for the expression profiling and sequence amplification of *1Bx20* and *1By20* in both AS208 and LX987. (DOC)

## References

[1] Wrigley CW. Giant proteins with flour power. Nature. 1996; 381: 738–739. 8657274 10.1038/381738a0

[2] Majoul T, Bancel E, Triboi E, Ben HJ, Branlard G. Proteomic analysis of the effect of heat stress on hexaploid wheat grain. Charaterization of heat responsive proteins from non-prolamins fraction. Proteomics. 2004; 4: 505–513. 14760723 10.1002/pmic.200300570

[3] Metakovsky EV, Novoselskaya AY, Sozinov AA. Genetic analysis of gliadin components in winter wheat using two-dimensional polyacrylamide gel electrophoresis. Theor Appl Genet. 1984; 69: 31–37. 10.1007/BF00262533 24253621

[4] Payne PI. Genetics of wheat storage proteins and the effect of allelic variation on bread making quality. Plant Physiol. 1987; 38: 141–153.

[5] Ma W, Apples R, Bekes F, Larroque O, Morell MK, Gale KR. Genetic characterisation of dough rheological properties in a wheat doubled haploid population: additive genetic effects and epistatic interactions. Theor Appl Genet. 2005; 111: 410–422. 15965651 10.1007/s00122-005-2001-0

[6] Rasheed A, Xia X, Yan Y, Appels R, Mahmood T, He Z. Wheat seed storage proteins: Advances in molecular genetics, diversity and breeding applications. J Cereal Sci. 2014; 60: 11–24.

[7] Branlard G, Dardevet M. Diversity of grain protein and bread wheat quality. Correlation between high molecular weight subunits of glutenin and flour quality characteristics. Cereal Sci. 1985; 3: 345–353.

[8] Payne PI, Holt LM, Krattiger AF, Carrillo JM. Relationships between seed quality characteristics and HMW glutenin subunit composition determined using wheat grown in Spain. J Cereal Sci. 1988; 7: 229–235.

[9] He ZH, Liu L, Xia XC, Liu JJ, Pena RJ. Composition of HMW and LMW glutenin subunits and their effects on dough properties, pan bread, and noodle quality of Chinese bread wheats. Cereal Chem. 2005; 82: 345–350.

[10] Payne PI, Corfield KG. Subunit composition of wheat glutenin proteins, isolated by gel filtration in a dissociating medium. Planta. 1979; 145: 83–88. 10.1007/BF00379931 24317568

[11] Payne PI, Genetics of wheat storage proteins and the effect of allelic variation on breadmaking quality. Annual Review Physiol. 1987; 38: 141–153.

[12] Payne PI, Holt LM, Jackson EA, Law CN. Wheat storage proteins: Their genetics and their potential for manipulation by plant breeding. Biological Sci. 1984; 304: 359–371.

[13] Mackie AM, Lagudah ES, Sharp PJ, Lafiandra D. Molecular and biochemical characterization of HMW glutenin subunits from *T*. *tauschii* and the D genome of hexaploid wheat. J Cereal Sci. 1996; 23: 213–225.

[14] D’Ovidio R, Masci S. The low-molecular-weight glutenin subunits of wheat gluten. J Cereal Sci. 2004; 39: 321–339.

[15] Juhasz A, Larroque OR, Tamas L, Hsam S L, Zeller FJ, Bekes F, et al Bankuti 1201–an old Hungarian wheat variety with special storage protein composition. Theor Appl Genet. 2003; 107: 697–704. 12750774 10.1007/s00122-003-1292-2

[16] Pang BS, Zhang XY. Isolation and molecular characterization of high molecular weight glutenin subunit genes *1Bx13* and *1By16* from hexaploid wheat. J Integrative Plant Bio. 2008; 50; 329–337. 10.1111/j.1744-7909.2007.00573.x 18713365

[17] Yan YM, Jiang Y, An XL, Pei YH, Zhang YZ, Wang AL, et al Cloning, expression and functional analysis of HMW glutenin subunit *1By8* gene from Italy pasta wheat (*Triticum turgidum* L.ssp. *durum*). J Cereal Sci. 2009; 50: 398–406.

[18] Shewry PR, Halford NG, Tatham AS. The high molecular weight subunits of wheat glutenin. J Cereal Sci. 1992; 15; 105–120.

[19] Shewry PR, Gilbert SM, Savage AWJ, Tatham AS, Belton PS, Wellner N, et al Sequence and properties of HMW subunit 1Bx20 from pasta wheat (*Triticum durum*) which is associated with poor end use properties. Theor Appl Genet. 2003; 106; 744–750. 12596005 10.1007/s00122-002-1135-6

[20] Li W, Wan Y, Liu Z, Liu K, Liu X, Li B, et al Molecular characterization of HMW glutenin subunit allele 1Bx14: further insights into the evolution of *Glu-B1-1* alleles in wheat and related species. Theor Appl Genet. 2004; 109: 1093–1104. 15290043 10.1007/s00122-004-1726-5

[21] Li YW, An XL, Yang R, Guo XM, Yue GD, Fan RC, et al Dissecting and enhancing the contributions of high-molecular-weight glutenin subunits to dough functionality and bread quality. Mol Plant. 2015; 8; 332–334. 25680778 10.1016/j.molp.2014.10.002

[22] He G.Y, Rooke L, Bekes F, Gras P, Tatham AS, Fido R, et al Transformation of pasta wheat (*Triticum turgidum* L. Var. durum) with high-molecular-weight glutenin subunit genes and modification of dough functionality. Mol Breed. 1999; 5: 377–386.

[23] Smith RL, Schweder ME, Barnett RD. Identification of glutenin alleles in wheat and triticale using PCR-generated DNA markers. Crop Sci. 1994; 34: 1373–1378.

[24] Ahmad M. Molecular marker-assisted selection of HMW glutenin alleles related to wheat bread quality by PCR-generated DNA markers. Theor Appl Genet. 2000; 101: 892–896.

[25] Butow BJ, Ma W, Gale KR, Cornish GB, Rampling L, Larroque O, et al Molecular discrimination of Bx7 alleles demonstrates that a highly expressed high-molecular-weight glutenin allele has a major impact on wheat flour dough strength. Theor Appl Genet. 2003; 107: 1524–1532. 13679992 10.1007/s00122-003-1396-8

[26] Ma W, Zhang W, Gale KR. Multiplex-PCR typing of high molecular weight glutenin alleles in wheat. Euphytica. 2003; 134: 51–60.

[27] Schwarz G, Sift A, Wenzel G, Mohler V. DHPLC scoring of a SNP between promoter sequences of HMW glutenin x-type alleles at the *Glu-D1* locus in wheat. J Agri Food Chem. 2003; 51: 4263–4267. 10.1021/jf0261304 12848495

[28] Schwarz G, Felsenstein FG, Wenzel G. Development and validation of a PCR-based marker assay for negative selection of the HMW glutenin allele *Glu-B1-1d* (*Bx-6*) in wheat. Theor Appl Genet. 2004; 109: 1064–1069. 15175854 10.1007/s00122-004-1718-5

[29] Lei ZS, Gale KR, He ZH, Gianibelli C, Larroque O, Xia XC, et al Y-type gene specific markers for enhanced discrimination of high-molecular weight glutenin alleles at the *Glu-B1* locus in hexaploid wheat. J Cereal Sci. 2006; 43: 94–101.

[30] Liu SX, Chao SM, Anderson JA. New DNA markers for high molecular weight glutenin subunits in wheat. Theor Appl Genet. 2008; 118: 177–183. 10.1007/s00122-008-0886-0 18797838

[31] Ragupathy R, Naeem HA, Reimer E, Lukow OM, Sapirstein HD, Cloutier S. Evolutionary origin of the segmental duplication encompassing the wheat *Glu-B1* encoding the overexpressed Bx7 (Bx7^OE^) high molecular weight glutenin subunit. Theor Appl Genet. 2008; 116: 283–296. 17985111 10.1007/s00122-007-0666-2

[32] Jin H, Yan J, Peña RJ, Xia XC, Morgounov A, Han LM, et al Molecular detection of high-and low-molecular-weight glutenin subunit genes in common wheat cultivars from 20 countries using allele-specific markers. Crop Pasture Sci. 2011; 62: 746–754.

[33] Weegels PL, Van de Pijpekamp AM, Graveland A, Hamer RJ, Schofield JD. Depolymerisation and re-polymerisation of wheat glutenin during dough processing. I. Relationships between glutenin macropolymer content and quality parameters. J Cereal Sci. 1996; 23: 103–111.

[34] Pirozi MR, Margiotta B, Lafiandra D, MacRitchie F. Composition of polymeric proteins and bread-making quality of wheat lines with allelic HMW-GS differing in number of cysteines. J Cereal Sci. 2008; 48: 117–122.

[35] Tatham AS, Miflin BJ, Shewry PR. The β-turn conformation in wheat gluten proteins: relationship to gluten elasticity. Cereal Chem. 1985; 62: 405–412.

[36] Flavell RB, Goldsborough AP, Robert LS, Schnick D, Thompson RD. Genetic variation in wheat HMW glutenin subunits and the molecular basis of bread-making quality. Nat Biotech. 1989; 7: 1281–1285.

[37] Tamas L, Gras PW, Solomon RG, Morell MK, Appels R, Bekes F. Chain extension and termination as a function of cysteine content and the length of the central repetitive domain in storage proteins. J Cereal Sci. 2002; 36: 313–325.

[38] Gupta RM, Masci S, Lafiandra D, Bariana HS, MacRitchie F. Accumulation of protein subunits and their polymers in developing grains of hexaploid wheats. J Exp Bot. 1996; 47: 1377–1385.

[39] Liu W, Zhang YZ, Gao X, Wang K, Wang SL, Zhang Y, et al Comparative proteome analysis of glutenin synthesis and accumulation in developing grains between superior and poor quality bread wheat cultivars. J Food Agri Envir. 2012; 92: 106–115. 10.1002/jsfa.4548 21815156

[40] Zhang X, Jin H, Zhang Y, Liu D, Li G, Xia X, et al Composition and functional analysis of low-molecular-weight glutenin alleles with Aroona near-isogenic lines of bread wheat. BMC Plant Bio. 2012; 12: 243. 23259617 10.1186/1471-2229-12-243 PMC3562532

[41] Barro F, Rooke L, Bekes F, Gras P, Tatham AS, Fido R, et al Transformation of wheat with high molecular weight subunit genes results in improved functional properties. Nat Biotech. 1997; 15: 1295–1299. 10.1038/nbt1197-1295 9359115

[42] Barro F, Barceló P. Functional properties of flours from field grown transgenic wheat lines expressing the HMW glutenin subunit *1Ax1* and *1Dx5* genes. Mol Breed. 2003; 12: 223–229.

[43] Altpeter Fredy, Juan CP, Wieser Herbert. Stable expression of *1Dx5* and *1Dy10* high-molecular-weight glutenin subunit genes in transgenic rye drastically increases the polymeric glutelin fraction in rye flour. Plant Mol Bio. 2004; 54: 783–792. 15604651 10.1007/s11103-004-0122-5

[44] Anderson OD, Bekes F, Gras PW, Kuhl JC, Tam A. Use of a bacterial expression system to study wheat HMW glutenins and the construction of synthetic HMW glutenin genes In: Wrigley C. W. (Ed.), Proceedings of the Sixth International Gluten Workshop. Royal Australian Chemical Institute, Cereal Chemistry Division, North Melbourne, Australia, 1996; pp 195–198.

[45] Békés F, Gras PW, Anderssen RS, Appels R. Quality traits of wheat determined by small-scale dough testing methods. Aust J Agri Res. 2001; 52: 1325–1338.

[46] Dowd C, Bekes F. Large-scale expression and purification of HMW glutenin subunits. Protein Expre and Purif. 2002; 25: 97–104. 10.1006/prep.2001.1614 12071704

[47] Yan Y, Hsam SLK, Yu SLK, Jiang Y, Ohtsuka I, Zeller FJ. HMW and LMW gluten in alleles among putative tetraploid and hexaploid *T*. *spelta* progenitors. Theor Appl Genet. 2003; 107: 1321–1330. 13679994 10.1007/s00122-003-1315-z

[48] Deng ZY, Tian JC, Sun GX. Influence of high molecular weight glutenin subunit substitution on rheological behavior and bread-baking quality of near-isogenic lines developed from Chinese wheats. Plant Breed. 2005; 124: 428–431.

[49] Zhao XC, Sharp PJ. An improved 1-D SDS-PAGE method for the identification of three bread wheat ‘Waxy’ proteins. J Cereal Sci. 1996; 23: 191–193.

[50] Gao LY, Ma WJ, Chen J, Wang K, Li J, Wang S, et al Characterization and comparative analysis of wheat high molecular weight glutenin subunits by SDS-PAGE, RP-HPLC, HPCE, and MALDI-TOF-MS. J Agri Food Chem. 2010; 58: 2777–2786. 10.1021/jf903363z 20146422

[51] Burnouf T, Bietz JA. Reversed-phase high-performance liquid chromatography of reduced glutenin, a disulfide-bonded protein of wheat endosperm. J Chromatography. 1984; 299: 185–199.

[52] AACC, 1995 Approved Methods of the American Association of Cereal Chemists, 9th Edition American Association of Cereal Chemists, Inc, St. Paul, MN.

[53] Arbeitsgemeinschaft Getreideforschung, 1994. Standard-Methoden fu¨er Getreide, Mehl und Brot, Verlag Moritz, Detmold.

[54] Shen XY, Yan J, Chen XM, Zhang Y, Li HL, Wang DS, et al Relationship of mixograph parameters with farinograph and extensograph parameters, and bread-making quality traits. Acta Agro Sin. 2010; 36: 1037–1043.

[55] Wang SL, Cao M, Yu ZT, Shen XX, Li N, Ma WJ, et al Molecular mechanisms of HMW glutenin subunits from 1S^l^ genome positively affecting wheat bread-making quality. PLoS ONE. 2013; 8: e58947. 23593125 10.1371/journal.pone.0058947 PMC3617193

[56] Lawrence GJ, Macritchie F, Wrigley CW. Dough and baking quality of wheat lines deficient in glutenin subunits controlled by the *Glu-A1*, *Glu-B1* and *Glu-D1* loci. J Cereal Sci. 1988; 7: 109–112.

[57] Yang Y, Li S, Zhang K, Dong Z, Li Y, An X, et al Efficient isolation of ion beam-induced mutants for homoeologous loci in common wheat and comparison of the contributions of *Glu-1* loci to gluten functionality. Theor Appl Genet. 2014; 127: 359–372. 10.1007/s00122-013-2224-4 24212587

[58] Upelnick VP, Novoselskaya AY, Sutka J, Galiba G, Metakovsky EV. Genetic variation at storage protein-coding loci of common wheat (cv ‘Chinese Spring) induced by nitrosoethylurea and by the cultivation of immature embryos *in vitro*. Theor Appl Genet. 1995; 90: 372–379. 24173927 10.1007/BF00221979

[59] Beshkova N, Ivanov P, Ivanova I. Further evidence for glutenin modifications in winter wheat (*Triticum aestivum* L.) induced by somaclonal variation. Biotechnol & Biotechnol Eq. 1998; 12(2): 53–57.

[60] Hammond SM, Caudy AA, Hannon GJ. Post-transcriptional gene silencing by double stranded RNA. Nature. 2001; 2: 110–119. 10.1038/35052556 11253050

[61] Bird A. DNA methylation patterns and epigenetic memory. Genes Dev. 2002; 16: 6–21. 11782440 10.1101/gad.947102

[62] Baylin SB. DNA methylation and gene silencing in cancer. Nat Clin Pract Oncol. 2005; 2: S4–S11. 16341240 10.1038/ncponc0354

[63] Richardson T, Thistleton J, Higgins TJ, Howitt C, Ayliffe M. Efficient *Agrobacterium* transformation of elite wheat germplasm without selection. Plant Cell Tiss Organ Cult. 2014; 119: 647–659.

[64] Ishida Y, Tsunashima M, Hiei Y, Komari Y. Wheat (Triticum aestivum L.) transformation using immature embryos Wang Kan (ed.), *Agrobacterium Protocols*: *Volume 1*, Methods in Molecular Biology, vol. 1223, Springer Science+Business Media New York, 2015; pp 189–198. 10.1007/978-1-4939-1695-5_15 25300841

[65] Ye XG. Development and application of plant transformation techniques. J Integrative Agri. 2015; 14: 411–413.

[66] Tao LL, Yin GX, Du LP, Shi ZY, She MY, Xu HJ, et al Improvement of plant regeneration from immature embryos of wheat infected by *Agrobacterium tumefaciens*. Agri Sci China. 2011; 10: 317–326.

[67] Yin GX, Wang YL, She MY, Du LP, Xu HJ, Ma JX, et al Establishment of a highly efficient regeneration system for the mature embryo culture of wheat. Agri Sci China. 2011; 10: 9–17.

