# Supplementary material for: Comprehensive Identification and Bread-Making Quality Evaluation of Common Wheat Somatic Variation Line AS208 on Glutenin Composition
Source: PLoS One. 2016 Jan 14;11(1):e0146933. doi: 10.1371/journal.pone.0146933 (PMC4713059; doi:10.1371/journal.pone.0146933)
Supplement: S1 Fig — In the second generation derived from tissue culture (a), a plant was found to be missing 1Bx20 and 1By20 (sample 5). The plants from sample 5 appeared different bands for the composition of HMW-GS, some with and some without 1Bx20 and 1By20 in the third generation (b). Stable line AS208 was obtained from sample 5 in the fourth generation, in which the two bands of 1Bx20 and 1By20 were missed completely. WT stands for LX987. (DOC) [file pone.0146933.s001.doc]

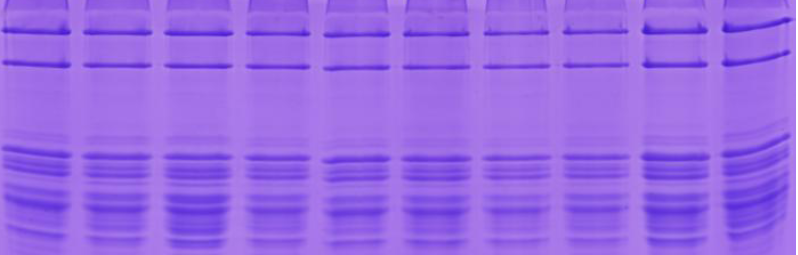


**c**

**S1 Fig.** **Screening of wheat somatic variation mutants at the *Glu-B1* locus in the tissue culture offspring of LX987 by SDS-PAGE.** In the second generation derived from tissue culture (a), a plant was found to be missing 1Bx20 and 1By20 (sample 5). The plants from sample 5 appeared different bands for the composition of HMW-GS, some with and some without 1Bx20 and 1By20 in the third generation (b). Stable line AS208 was obtained from sample 5 in the fourth generation, in which the two bands of 1Bx20 and 1By20 were missed completely. WT stands for LX987.
