# Supplementary material for: Comprehensive Identification and Bread-Making Quality Evaluation of Common Wheat Somatic Variation Line AS208 on Glutenin Composition
Source: PLoS One. 2016 Jan 14;11(1):e0146933. doi: 10.1371/journal.pone.0146933 (PMC4713059; doi:10.1371/journal.pone.0146933)
Supplement: S2 Fig — 1Dx2 and 1Dy12, as well as LMW-GSs genes expressed stably in AS208 from the 11th day post anthesis (lane 3), while 1Bx20 and 1By20 were not expressed at any point during the whole period of grain development. 1–10 stand for the samples collected at 5, 8, 11, 13, 17, 19, 21, 23, 26, and 29th day post anthesis, respectively. (DOC) [file pone.0146933.s002.doc]

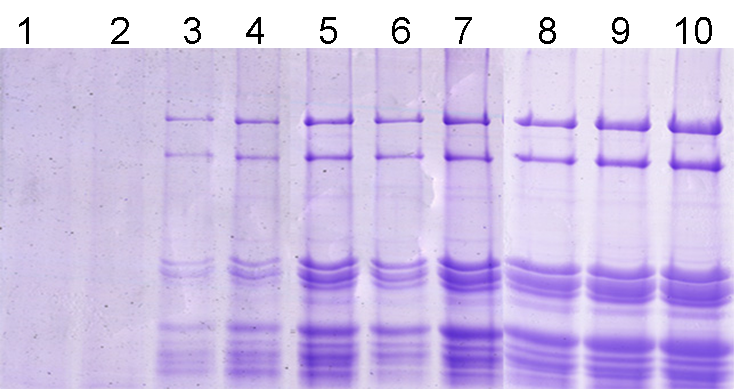


**S2 Fig.** **Expression patterns of genes encoding glutenin subunits in AS208 at different stages during grain development assessed by SDS-PAGE.** *1Dx2* and *1Dy12*, as well as *LMW-GSs* genes expressed stably in AS208 from the 11^th^ day post anthesis (lane 3), while *1Bx20* and *1By20* were not expressed at any point during the whole period of grain development. 1-10 stand for the samples collected at 5, 8, 11, 13, 17, 19, 21, 23, 26, and 29th day post anthesis, respectively.
