# Supplementary material for: Comprehensive Identification and Bread-Making Quality Evaluation of Common Wheat Somatic Variation Line AS208 on Glutenin Composition
Source: PLoS One. 2016 Jan 14;11(1):e0146933. doi: 10.1371/journal.pone.0146933 (PMC4713059; doi:10.1371/journal.pone.0146933)
Supplement: S3 Fig — 1Bx20 was not detected in the genome of AS208 by the marker (nothing was amplified). However, 1Bx20 was detected from genomic DNA of LX987 by the markers (a 216-bp fragment was amplified). M represents a DL2000 DNA marker. (DOC) [file pone.0146933.s003.doc]

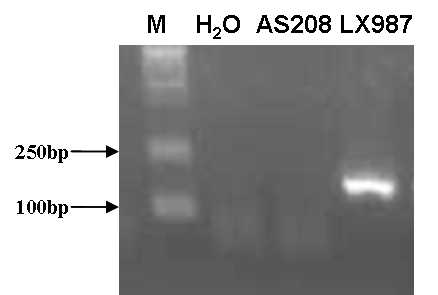


**S3 Fig.** **Detection of *1Bx20* in AS208 and LX987 with the M1 and M2 PCR markers.** *1Bx20* was not detected in the genome of AS208 by the marker (nothing was amplified). However, *1Bx20* was detected from genomic DNA of LX987 by the markers (a 216-bp fragment was amplified). M represents a DL2000 DNA marker.
