# Supplementary material for: Comprehensive Identification and Bread-Making Quality Evaluation of Common Wheat Somatic Variation Line AS208 on Glutenin Composition
Source: PLoS One. 2016 Jan 14;11(1):e0146933. doi: 10.1371/journal.pone.0146933 (PMC4713059; doi:10.1371/journal.pone.0146933)
Supplement: S1 Table — (DOC) [file pone.0146933.s004.doc]

**S1 Table.** **The PCR primers used in this study for the expression profiling and sequence amplification of *1Bx20* and *1By20* in both AS208 and LX987**.

| **Purpose** | **Name** | **Sequence** |
| --- | --- | --- |
| Expression analysis and genomic detection for *1Bx20* | M1 | TGAGCGCGAGCTCCGGAAG |
|  | M2 | AAGGCGTAGTCTCGCTGGGG |
| Promoter amplification for *1Bx20* | Px1 | ATGGCGTGACCAAGCGATAAG |
|  | Px2 | CAGGTTGCTGCTCGTATTGTCT |
| Promoter amplification for *1By20* | Py1 | GTTGGACGATGGGAGATGAAAG |
|  | Py2 | GTGGTCTCGCCAGGGTAGAAGG |
| Open reading frame (ORF) amplification for *1Bx20* | Bx1 | ATGGCTAAGCGGC/TTA/GGTCCTCTTTG |
|  | Bx2 | CTATCACTGGCTG/AGCCGACAATGC |
| Open reading frame(ORF) amplification for *1By20* | By1 | ATGGCTAAGCGGTTAGTCCT |
|  | By2 | GCTGCAGAGAGTTCTATC |
